# Reassessing the role of basal cortisol in ACTH stimulation testing for canine hypoadrenocorticism: insights from a large UK referral and first-opinion dataset

**DOI:** 10.3389/fvets.2025.1686045

**Published:** 2025-10-16

**Authors:** Armando C. Sánchez-Lara, Michael E. Herrtage, Tim L. Williams, Helen Evans, Abigail Stout, Andre Kortum

**Affiliations:** ^1^Department of Veterinary Medicine, School of Biological Sciences, Queen’s Veterinary School Hospital, The University of Cambridge, Cambridge, United Kingdom; ^2^NationWide Specialist Laboratories, Eastern Counties Leather Industrial Estate, Pampisford, Cambridge, United Kingdom

**Keywords:** basal cortisol, redundant pre-ACTH cortisol, canine hypoadrenocorticism, post-ACTH cortisol, Addison’s disease

## Abstract

**Introduction:**

Canine hypoadrenocorticism (HA) is characterized by glucocorticoid (and often mineralocorticoid) deficiency and typically requires ACTH stimulation testing for diagnosis. A basal (pre-ACTH) cortisol >55 nmol/L is widely used to exclude HA, but prior studies were limited to referral populations. Its utility in first-opinion practice remains unclear.

**Objectives:**

To evaluate the diagnostic performance of basal cortisol for identifying HA, and its added value alongside post-ACTH cortisol, using UK laboratory data predominantly from first-opinion practices and the remainder from referral centers.

**Materials and methods:**

Retrospective analysis of 1,017 ACTH stimulation tests (January 2019–April 2023) from a UK veterinary diagnostic laboratory. After excluding cases tested for hypercortisolism, 878 cases remained: 170 from a referral center (RC) with full clinical history and 708 predominantly from first-opinion (FO) practices. Serum cortisol was measured by radioimmunoassay. HA was defined as post-ACTH cortisol ≤55 nmol/L. Diagnostic performance metrics were calculated for basal cortisol cut-offs of ≤55 and ≤22 nmol/L.

**Results:**

HA prevalence was 8.4% in RC, 4.4% in FO, and 5.1% in combined groups (RC + FC). RC group basal cortisol ≤55 nmol/L showed a high sensitivity (93%) and NPV (99%), but low specificity (77%), and PPV (25%). Reducing the cut-off to ≤22 nmol/L improved specificity (92%) and PPV (50%), maintaining sensitivity (93%) and NPV (99%). However, when assessing the FO group only or when combined with RC (RC + FO), the ≤22 nmol/L cut-off sensitivity was slightly reduced (90 and 91%, respectively). Receiver operating characteristic analysis yielded areas under the curve of 0.93–0.97 for basal cortisol and 1.0 for post-ACTH cortisol across all datasets. Pre-ACTH cortisol provided no additional diagnostic value when post-ACTH cortisol was known.

**Conclusion:**

Basal cortisol >55 nmol/L is a reliable rule-out test for canine HA in both referral and first-opinion settings, at prevalences lower than seen in referral practices. However, when an ACTH stimulation test is conducted, post-ACTH cortisol alone provides perfect diagnostic accuracy, rendering pre-ACTH cortisol redundant. These findings support omitting pre-ACTH cortisol in routine ACTH stimulation testing to streamline diagnostics and reduce costs without compromising diagnostic performance.

## 1 Introduction

Canine hypoadrenocorticism (HA) is characterized by glucocorticoid (and usually mineralocorticoid) insufficiency (1). Dogs with primary hypoadrenocorticism typically present with vague and variable clinical signs, most commonly including lethargy, gastrointestinal disturbances (vomiting, diarrhea, anorexia, and, less commonly, hematemesis and melena), weight loss, and occasionally collapse. These signs are often nonspecific and can mimic other systemic illnesses, contributing to the diagnostic challenge. Electrolyte abnormalities such as hyponatraemia and hyperkalaemia are characteristic of combined glucocorticoid and mineralocorticoid deficiency but are not invariably present. Dogs with eunatraemic, eukalaemic hypoadrenocorticism, in which glucocorticoid deficiency occurs without overt electrolyte derangements, may present with similar nonspecific signs but maintain normal sodium and potassium concentrations (2, 3).

The diagnosis is conventionally confirmed by assessment of serum cortisol concentrations before and after administration of adrenocorticotrophic hormone (ACTH stimulation testing) (4). Basal cortisol concentrations have shown a high sensitivity (99.4–100%) and moderate specificity (63.3–78.2%) for the diagnosis of HA using a cut off ≤55 nmol/L according to three studies (5–7). However, the studies available investigated cases from referral centers and may not be representative of cases tested for HA in first opinion practice.

Basal cortisol (also known as pre-ACTH or random cortisol) concentration can be used as a screening tool to exclude HA; however, its positive and negative predictive values are less well-defined, and these can be significantly influenced by the disease prevalence in the tested population. Moreover, the cortisol assay currently in use, a radioimmunoassay, differs from the chemiluminescent immunoassay previously evaluated in UK dogs with HA (5). Although the performance of radioimmunoassays has been assessed in U. S. populations (6, 7) geographical differences in disease prevalence may affect the generalizability of these findings.

This study therefore aims to retrospectively analyze cases with suspected or confirmed HA from January 2019–2023 to establish the sensitivity, specificity, negative and positive predictive value, and likelihood ratio of pre-ACTH serum cortisol concentrations (basal cortisol) for the diagnosis of HA and its diagnostic utility when submitted alongside post-ACTH serum cortisol concentrations using a large dataset of diagnostic test results from a diagnostic laboratory with referral and primarily first opinion populations.

## 2 Materials and methods

This retrospective study analyzed all available pre- and post-ACTH stimulation cortisol results from serum samples submitted to a UK veterinary diagnostic laboratory (VDL) between January 2019 and April 2023, by both first-opinion and referral practices. VDL cortisol analysis was performed using radioimmunoassay (Beckman Coulter). Cortisol results were obtained by searching the laboratory electronic and paper-based archive. Clinical information from VDL data was limited to patient signalment and notes referring to trilostane treatment when blood samples were submitted for hypercortisolism monitoring purposes. A detailed clinical history was collected from a single referral center that submitted blood samples to VDL during the same period.

Three datasets were analyzed: a single referral center cohort with complete clinical histories (RC group), the VDL dataset representing predominantly first-opinion practice submissions without RC cases (FO group), and the combined dataset including both referral center and VDL submissions (RC + FO).

Exclusion criteria for the FO group (VDL database) included results suspected of hypercortisolism identified by reported trilostane treatment, repeated ACTH stimulation tests, or biochemical findings suggestive of hypercortisolism (e.g., marked ALP elevation with mild to moderate ALT increases and normal total bilirubin). Submissions for pre-pill cortisol or trilostane monitoring were excluded. For the RC group, cases were excluded if testing was performed for suspicion or monitoring of hypercortisolism, or if the dog had received exogenous glucocorticoids within the 4 weeks before testing. Regarding inclusion criteria, all results from the VDL database related to cortisol submissions, ACTH stimulation tests, and complete adrenal analysis panels were included in the study. For the RC cohort, additional inclusion criteria required a full clinical history, hematology and biochemistry at admission, abdominal ultrasonography, and measurement of both pre- and post-ACTH cortisol concentrations. The RC group was evaluated separately to provide an accurate prevalence estimate in a referral population, where complete medical records were available and the indication for cortisol testing could be confirmed. Serum samples from this group were analyzed at the same VDL as those from the FO cohort during the same study period.

Hypoadrenocorticism cases were based on diagnosis defined by a post-ACTH cortisol concentration of ≤55 nmol/L (4–8) for both RC and FO groups. All samples with post-ACTH >55 nmol/L were regarded as non-HA, as per ACTH test.

The response to treatment for RC cases, defined as marked improvement or normalization of clinical signs (e.g., gastrointestinal, lethargy, hyporexia, polyuria and polydipsia) and abnormal biochemical markers including (electrolyte derangements and azotemia), was considered a secondary validation of diagnosis. Follow up duration of cases ranged from one to 8 months, after which cases were managed by the referring veterinarians, limiting further communication.

Data analyses were conducted using Prism 5 software (GraphPad, La Jolla, CA). The sensitivity, specificity, and likelihood ratios of pre-ACTH cortisol for detecting HA in dogs were calculated using two cut-off values: ≤55 and ≤22 nmol/L. Positive and negative predictive values were calculated based on three population prevalences, RC, FO, and RC + FO groups. Areas under the receiver operating characteristic (ROC) curve analysis were calculated for both pre- (basal cortisol) and post-ACTH cortisols. Statistical significance was defined as *p* < 0.05.

## 3 Results

**Figure 1:**
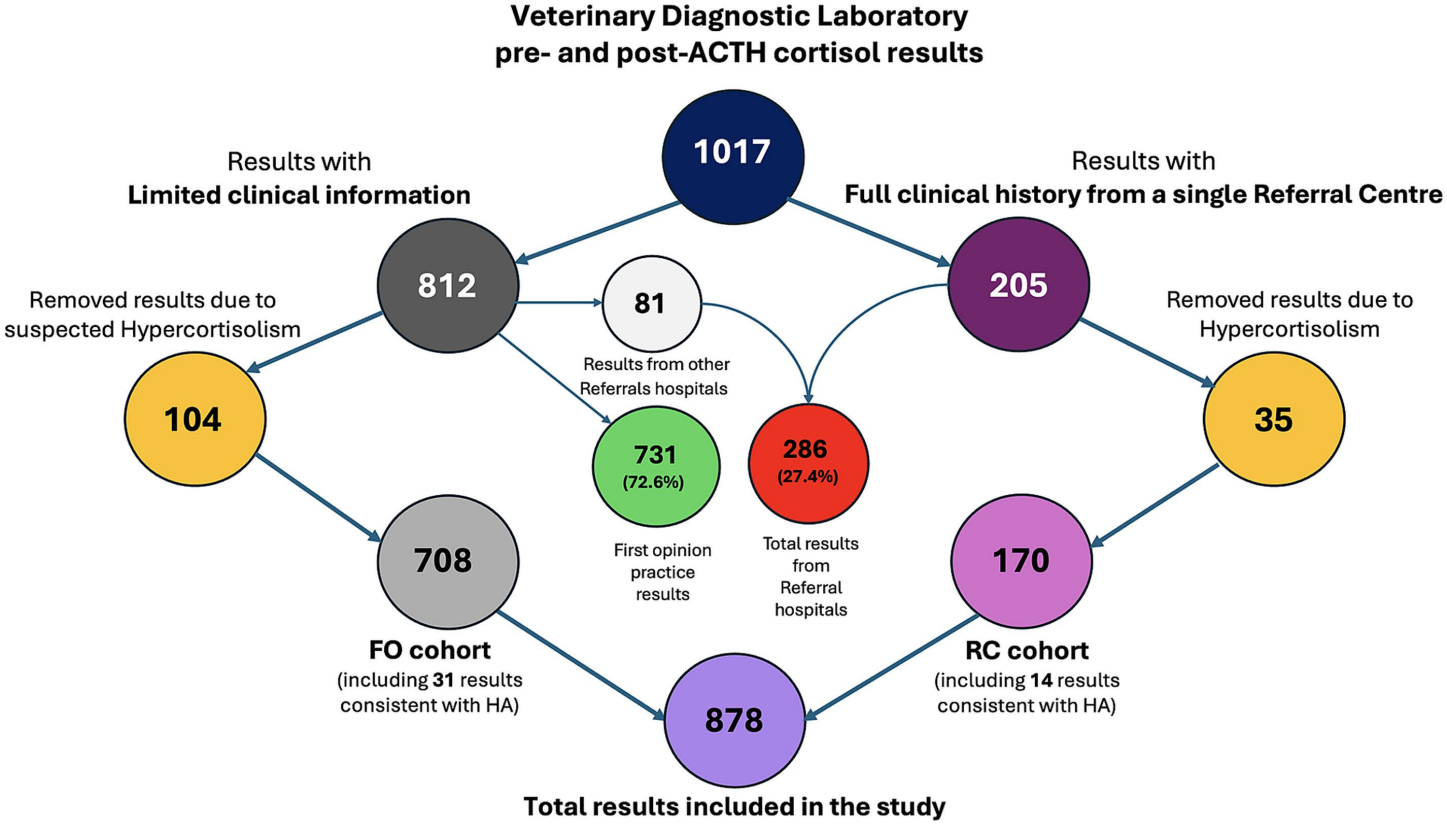
Flowchart illustrating the selection process of pre- and post-ACTH stimulation cortisol results from the Veterinary Diagnostic Laboratory archives between January 2019 and April 2023. A total of 1,017 results were collected (blue circle), with 27.4% (286/1,017) originating from referral centers (red circle) and 72.6% (731/1,017, green circle) from first-opinion practitioners. Among these, 205 (violet circle) results originated from a single referral center, where comprehensive clinical histories were available; the remaining 812 results (dark gray circle) had limited clinical information. Thirty-five (yellow right circle) of the 205 referral hospital cases were consistent with hypercortisolism and were excluded, leaving 170 results (RC group, pink circle) with detailed clinical histories. An additional 104 cases from the limited-history group (yellow right circle) were excluded due to suspected hypercortisolism, resulting in 708 cases for analysis (FO cohort, light gray circle). In total, 878 results were included in the final study population (Total results included, purple circle). HA, hypoadrenocorticism; FO, predominantly first-opinion group; RC, referral center group.

A total of 1,017 paired pre- and post-ACTH stimulation cortisol results were retrieved from the VDL archives, spanning January 2019 to April 2023 (Figure 1). Of these, 286 results (27.4%) were from referral centres, while the remaining 731 results (72.6%) originated from first opinion practice. Among the 286 results from referral centers, 205 originated from a single veterinary referral center, where complete clinical histories and diagnostic tests were available. The remaining 81 referral cases were contributed by other referral centers and lacked detailed historical information. Thirty-five cases from the single referral center with full clinical histories were excluded based on clinical and hormonal data consistent with canine hypercortisolism, resulting in a final subset of 170 cases included for analysis, referred to as RC group. From the rest of the VDL results 104 cases were similarly excluded for suspected canine hypercortisolism, leaving 708 eligible cases, including 627 originated from first opinion and 81 from referral practices, referred as FO group.

In total, 878 cortisol results were included in the final analysis, comprising 708 cases from the FO cohort and 170 cases from RC cohort. A total of 45 dogs with confirmed HA were obtained. The remaining 833 cases had post-ACTH cortisol concentrations >55 nmol/L and were therefore categorized as non-HA dogs, per ACTH test. Of the 45 HA cases, 31 were from the FO group and 14 from the RC cohort. Within the RC group, three dogs had isolated glucocorticoid deficiency (eunatraemic, eukalaemic hypoadrenocorticism), while the remaining 11 had combined glucocorticoid and mineralocorticoid deficiency (hyperkalaemic and/or hyponatraemic hypoadrenocorticism) and required both prednisolone and mineralocorticoid supplementation, administered as either fludrocortisone (*n* = 2) or desoxycorticosterone pivalate (*n* = 9). Response to treatment was notified in 11 of the 14 cases. Assessment of treatment response was not possible in two dogs with combined glucocorticoid and mineralocorticoid deficiency due to death secondary to gastrointestinal perforation attributed to primary HA, confirmed by endogenous ACTH. The remaining case, a dog with eunatraemic, eukalaemic hypoadrenocorticism, was classified as having a partial clinical response possibly related to adequacy of supplementation.

The diagnostic performance of pre-ACTH stimulation cortisol concentrations for diagnosis of HA was assessed using two cut-offs: ≤55 and ≤22 nmol/L (Table 1). In the RC group, a cortisol concentration ≤55 nmol/L yielded a sensitivity of 93% (95% CI, 69–100%), specificity of 77% (95% CI, 67–82%), positive predictive value (PPV) of 25% (95% CI, 13–36%), and negative predictive value (NPV) of 99% (95% CI, 98–100). Reducing the cut-off to ≤22 nmol/L maintained sensitivity at 93% but improved specificity to 92% (95% CI, 86–95%), with a corresponding increase in PPV from 25 to 50% (95% CI, 31–62%). The likelihood ratio also increased from 3.7 to 12. The prevalence of HA in the referral dataset was 8%.

**Table 1:** Diagnostic accuracy metrics of basal serum cortisol thresholds (≤55 and ≤22 nmol/L) for hypoadrenocorticism in dogs, evaluated in a referral center, predominantly first-opinion, and combined cohorts.

Pre-ACTH cortisol (nmol/L)	Dogs with HA	non-HA dogs, per ACTH test	Sensitivity % (95% CI)	Specificity % (95% CI)	NPV% (95% CI)	PPV% (95% CI)	Likelihood ratio	Prevalence %
RC group								
≤55	14	156	93 (69–100)	77 (67–82)	99.4 (98–100)	24.5 (13–36)	3.7	8.4
≤22			93 (69–100)	92 (86–95)	99.4 (98–100)	50 (30.8–62.2)	12	
FO group								
≤55	31	677	100 (89–100)	81 (77.4–83)	100 (99.4–100)	19 (13–25)	5.1	4.4
≤22			90.3 (75–97)	95.9 (94–97)	100 (99.4–100)	50 (37.6–62.4)	21.9	
RC + FO group								
≤55	45	833	97.8 (88–100)	80 (77–82)	99.9 (96.6–100)	20.7 (15.3–26)	4.7	5.1
≤22			91 (79–96)	95 (83–96)	99.9 (99.6–100)	50.6 (40.1–61)	19	

Sensitivity, specificity, positive and negative predictive values, likelihood ratios, and prevalence of serum cortisol concentrations measured ≤55 and ≤22 nmol/L before ACTH stimulation (basal cortisol) in dogs with hypoadrenocorticism and those with non-HA, per ACTH stim test. Data are presented from three cohorts: a referral center (RC group) with comprehensive clinical histories, a predominantly first-opinion practice with limited clinical histories (FO group), and a combined dataset (RC + FO group). HA, hypoadrenocorticism; 95% CI, 95% confidence intervals.

In the FO group, a cut-off of ≤55 nmol/L achieved 100% sensitivity (95% CI, 89–100) and 81% specificity (95% CI, 77–83%), with a PPV of 19% (95% CI, 13–25%) and NPV of 100% (95% CI, 99–100%). Using the stricter threshold of ≤22 nmol/L reduced sensitivity to 90% (95% CI, 75–97%) but improved specificity to 96% (95% CI, 94–87%) and increased the PPV to 50% (95% CI, 38–62%). The corresponding likelihood ratios were 5.1 and 21.9 for ≤55 and ≤22 nmol/L cut-offs, respectively. The prevalence of HA in this primarily first-opinion population was lower, at 4%.

When combining both datasets (RC + FP group), a threshold of ≤55 nmol/L resulted in a sensitivity of 98% (95% CI, 88–100%), specificity of 80% (95% CI, 77–82%), NPV of 99.9% (95% CI, 97–100%), and PPV of 21% (95% CI, 15–26%). At the ≤22 nmol/L threshold, sensitivity decreased to 91% (95% CI, 79–96%), while specificity increased to 95% (95% CI, 83–96%), with a PPV of 51% (95% CI, 40–61%) and NPV of 99.9% (95% CI, 99–100%). The likelihood ratio improved from 4.7 to 19 using the ≤55 and ≤22 nmol/L cortisol cut-offs, respectively, while the overall prevalence of HA in the combined dataset was 5.1%.

**Figure 2:**
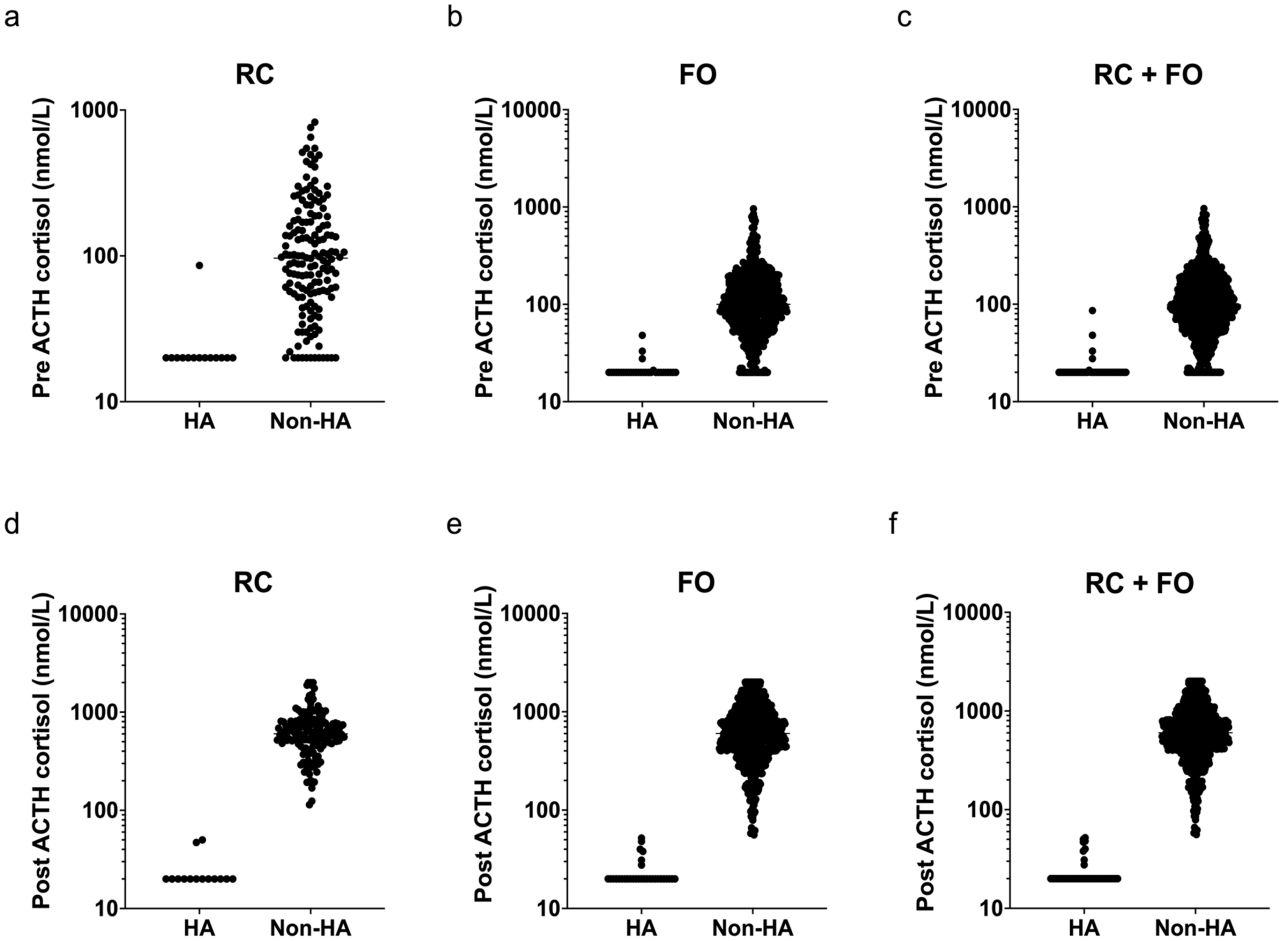
Scatter plots of pre-ACTH **(a–c)** and post-ACTH **(d–f)** serum cortisol concentrations (nmol/L) in dogs diagnosed with hypoadrenocorticism and those with non-HA, per ACTH stim test. Data are presented from three sources: **(a** and **d)**. a referral center with comprehensive clinical histories (RC), **(b** and **e)** a predominantly first-opinion practice with limited clinical histories (FO), and **(c** and **f)** a combined dataset (RC + FO). HA, hypoadrenocorticism; Non-HA, Non-hypoadrenocorticism, per ACTH test; FO, predominantly first-opinion group; RC, referral center group.

Scatter plots of pre- and post-ACTH stimulation serum cortisol concentrations revealed a clear separation between dogs with HA and non-HA dogs across all datasets (Figure 2). In both the RC (Figure 2a) and FO (Figure 2b) cohorts, dogs with HA consistently exhibited markedly lower pre-ACTH cortisol concentrations compared to those non-HA dogs, per ACTH test. The combined dataset (Figure 2c) maintained this distinction, with minimal overlap between groups. Similarly, post-ACTH cortisol concentrations (Figures 2d–f) demonstrated pronounced differences, with HA dogs showing uniformly low responses, often below the assay detection limit, in contrast to the broad distribution of post-stimulation cortisol concentrations observed in non-HA dogs’ cases. This pattern was consistent across RC, FO, and the combined dataset (RC + FO), supporting the diagnostic utility of both basal and stimulated cortisol concentrations for differentiating HA from other illnesses.

**Figure 3:**
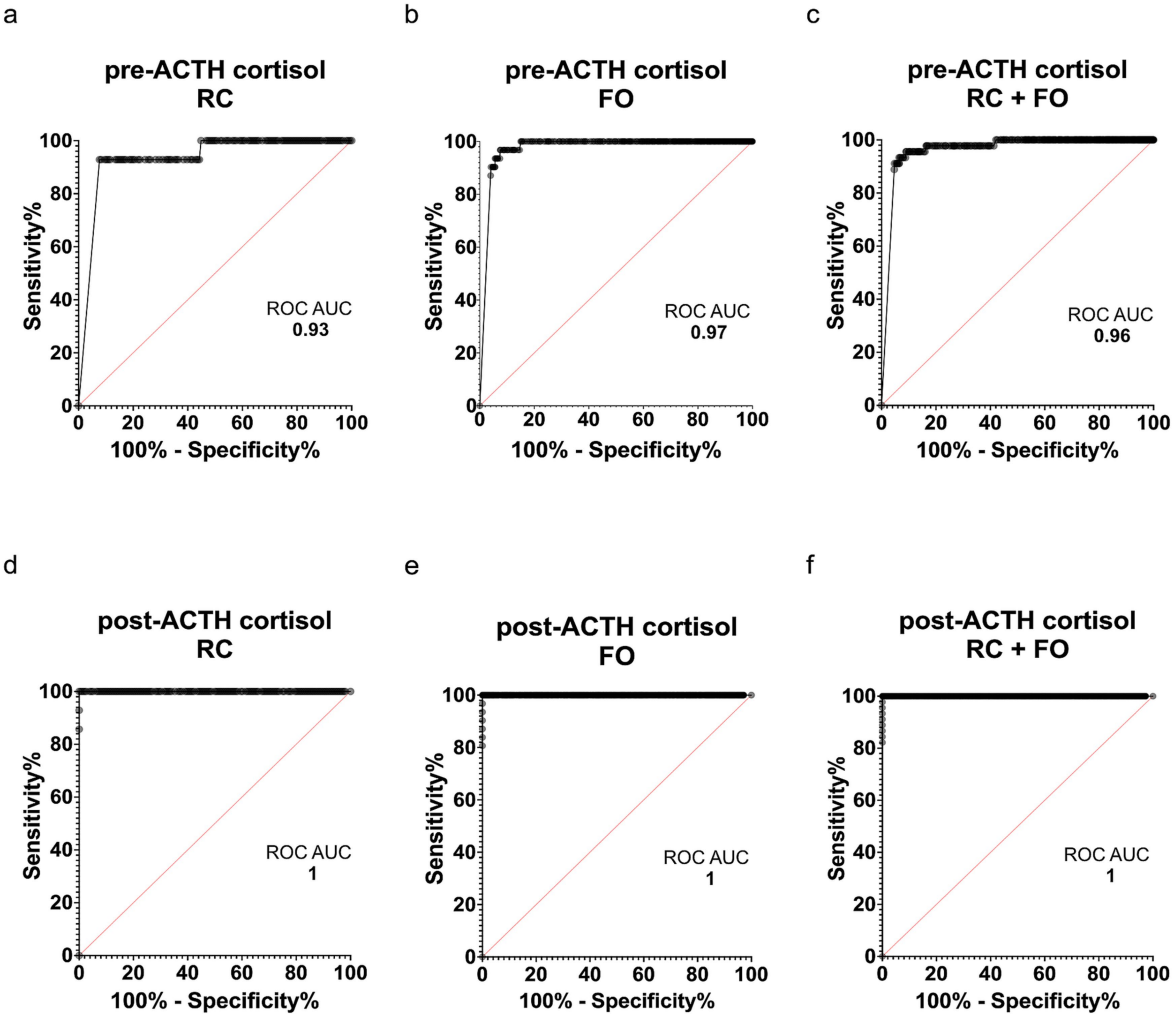
Receiver operating characteristic (ROC) curves illustrating the diagnostic performance of serum cortisol concentrations for hypoadrenocorticism in dogs. The diagonal line represents a non-informative test [area under the curve (AUC) = 0.5]. **(a–c)** Pre-ACTH cortisol ROC curves with AUCs of 0.93, 0.97, and 0.96 for the RC, FO, and combined data sets (RC + FO), respectively. **(d–f)** Post-ACTH cortisol ROC curves with an AUC of 1.0 across all datasets.

Receiver operating characteristic (ROC) curve analysis was used to assess the diagnostic performance of serum cortisol concentrations in differentiating dogs with HA from those with non-HA dogs (Figure 3). The area under the curve (AUC) for pre-ACTH cortisol was 0.93 (95% CI, 0.87–0.99) in the RC dataset, 0.97 (95% CI, 0.96–0.98) in the FO cohort, and 0.96 (95% CI, 0.94–0.98) in the combined dataset RC + FO (Figures 3a–c), indicating excellent but not perfect discriminative ability. In contrast, the AUC for post-ACTH cortisol was 1.0 (95% CI, 1–1) across all datasets (Figures 3d–f) and pre-ACTH cortisol did not alter interpretation when post-ACTH cortisol was ≤55 nmol/L.

## 4 Discussion

This study aimed to evaluate the diagnostic utility of pre- and post-ACTH stimulation serum cortisol concentrations in dogs with suspected HA across both referral and first-opinion settings. Using a large, retrospective dataset comprising 878 ACTH stimulation tests, we found that a basal serum cortisol concentration of ≤55 nmol/L demonstrated high sensitivity (97.8%) and negative predictive value (NPV; 99.9%) for the diagnosis of HA across all populations. However, basal cortisol concentrations lacked specificity and positive predictive value (PPV) implying a high rate of false-positive results. A lower basal cortisol concentration cut point of ≤22 nmol/L retained good diagnostic performance in the RC cohort compared to ≤55 nmol/L cut-off, as observed by Gold et al. (6), with the increased test specificity (from 77 to 92%) and PPV (from 24 to 50%) suggesting that a lower cut-off could be considered in referral population settings to increase suspicion of HA cases. Similar findings were also observed in the FO cohort and combined data (RC + FO). However, the sensitivity of pre-ACTH serum cortisol concentrations for diagnosis of HA in the FO cohort dropped from 100 to 90.3% when using the lower basal cortisol concentration cut off (≤22 nmol/L) suggesting differences in the populations assessed between the present study and in contrast to the findings of previous studies (5–7). As expected, post-ACTH cortisol concentrations provided perfect diagnostic accuracy (AUC = 1.0 in all cohorts) with most of the RC HA cases showing a positive clinical response to cortisol and, where indicated, mineralocorticoid supplementation, reaffirming post-ACTH cortisol ≤55 nmol/L as the gold standard for diagnosing HA. While not all cases could be monitored longitudinally due to loss to follow up or in-hospital death attributed to HA in two hyperkalaemic hyponatraemic HA cases, the available clinical outcomes align strongly with the diagnostic findings.

Despite differences in population characteristics, the diagnostic performance of basal cortisol was remarkably consistent across both referral and first-opinion data. In the RC cohort, where the prevalence of HA was 8.4%, the PPV for basal cortisol at the ≤55 nmol/L cut-off was 24.5%, and NPV was 99.4%. In contrast, the FC cohort, with a lower prevalence of 4.4%, yielded a PPV of 19% and an almost identical NPV of 100%. These findings underscore that while disease prevalence does influence PPV, the consistently high NPV at this cut off supports the continued use of basal serum cortisol concentrations as an effective rule-out test in both general practice and referral settings. The higher prevalence observed in the RC dataset is likely attributable to more selective testing or a true increase in disease prevalence due to referral of diagnostically complex or atypical cases. Dogs with HA often present with non-specific clinical signs and unremarkable findings on routine bloodwork and imaging, particularly in the case of eunatraemic eukalaemic hypoadrenocorticism which lack electrolyte abnormalities (9, 10). As such, the relative incidence of these cases may be increased in referral compared to first-opinion practice settings. These results align with prior observations and validate the importance of considering context when interpreting prevalence and diagnostic test performance (11).

Previous studies evaluating basal cortisol for HA (5–7) were all conducted in predominantly referral populations, potentially limiting their generalization. Our RC data demonstrated similar test characteristics to those reports, supporting the external validity of referral-center-derived estimates. Notably, Bovens et al. (5) reported that a serum cortisol concentration of <55 nmol/L was 99.4% sensitive and 37.3% specific for HA when using the chemiluminescent immunoassay, and similarly, Gold et al. (6) found a 99.4% sensitivity and 68.6% specificity for HA when using a ≤55 nmol/L cut point (determined by radioimmunoassay) in a U. S. population. Within our RC cohort, a serum cortisol concentration of ≤55 nmol/L (derived using the radioimmunoassay) was 93% sensitive and 77% specific for HA. Compared to Gold et al. (18.4%) PPV in our combined dataset (20.7%) was similar at the same cortisol threshold (≤55 nmol/L), and NPV remained consistently high in both studies (>99%), reinforcing the utility of basal serum cortisol concentrations for exclusion rather than confirmation of disease. The reduced sensitivity observed in the present study compared to previous reports was due to a single eunatraemic eukalaemic HA case with non-specific presentation and equivocal response to treatment.

In addition, this study also evaluated whether measurement of basal cortisol concentrations during ACTH stimulation testing offered any additional diagnostic benefit compared to post-ACTH cortisol alone. Our data show that measurement of pre-ACTH serum (basal) cortisol concentrations does not enhance the ACTH stimulation test’s sensitivity, specificity, PPV, or NPV and the AUC may vary according to the populations assessed (RC group, AUC = 0.93 vs. FO group, AUC = 0.97). This is contrary to other dynamic blood tests such as bile acid stimulation tests where fasting and post prandial bile acids are complementary in the assessment of hepatic function (12, 13). Other studies evaluating a single time point basal cortisol: ACTH ratio have shown overlap between HA and non-HA cases, and this approach has not been demonstrated to be superior to post-ACTH cortisol alone (14).

In the present study, post-ACTH cortisol alone was sufficient for definitive diagnosis of HA, with no overlap between diseased and non-diseased dogs, and an AUC of 1.0 across all datasets. The continued inclusion of pre-ACTH cortisol in routine ACTH stimulation testing thus merits re-evaluation. In addition to offering no additive diagnostic value, the inclusion of a pre-ACTH cortisol measurement alongside the post-ACTH cortisol result introduces practical and clinical drawbacks. Collecting, labeling, submitting, and processing two separate blood samples per patient increases the potential for pre-analytical error, particularly sample mislabeling, as documented in human medical settings (15, 16). Such errors may delay diagnosis and treatment, especially when results are discrepant or unexpected (e.g., pre- ACTH cortisol >55 nmol/L and post-ACTH cortisol <55 nmol/L). Furthermore, submission of an additional sample increases laboratory costs, imposes a greater logistical burden on clinical staff, and may contribute to unnecessary patient blood sampling and stress, particularly in non-critical cases where recent general blood analyses are already available. In these scenarios, synthetic ACTH formulations can be administered intramuscularly, allowing for a simplified protocol involving only two interventions: a single intramuscular ACTH injection followed by a post-ACTH blood sample 1 h later (17). This approach eliminates the need for a basal blood sample and the placement of an intravenous catheter. Discontinuing the routine measurement of pre-ACTH cortisol could streamline workflow, reduce resource utilization, and eliminate a redundant data point without compromising diagnostic accuracy.

Limitations of this study are primarily related to its retrospective design and the limited historical and other clinical information available for review in the FO cohort. This incomplete data may have influenced estimates of sensitivity, specificity, disease prevalence, and, consequently, the predictive values derived from this dataset. Nevertheless, a rigorous collation process was undertaken to minimize inappropriate inclusion of cases and bias: each case was carefully reviewed using all available data to exclude cases where testing was performed for a suspicion or monitoring of canine hypercortisolism, including those with serial test results, or those with biochemical profiles indicative of hypercortisolism or medical records referencing trilostane therapy. Additionally, while the FO cohort predominantly comprised first-opinion practice submissions, it did include 81 results (11.4%) from referral centers. These contributed to only five of the 31 HA cases identified in the cohort. Given the low proportion of these cases and the rarity of the disease in the overall population, it was considered appropriate to retain them in the FO analysis. Nonetheless, despite its limitations, the study provides meaningful guidance for first-opinion clinicians, with 88.6% of cases originating from primary care settings. Finally, we cannot exclude the possibility that critical illness may have influenced cortisol levels, particularly in the FO cohort; however, such cases do not typically show post-ACTH cortisol concentrations <55 nmol/L (8).

In conclusion, this study confirms the high sensitivity and NPV of basal serum cortisol concentrations for exclusion of HA at previously established diagnostic cut points, regardless of clinical setting (population from first opinion or referral centers) or disease prevalence. Nonetheless, basal cortisol concentrations below these cut offs occur commonly in dogs without HA and should not be relied upon for definitive diagnosis. When an ACTH stimulation test is conducted, post-ACTH cortisol alone provides perfect diagnostic accuracy, rendering pre-ACTH cortisol redundant. These findings therefore support a refined approach to diagnostic testing, advocating for the selective use of basal serum cortisol assessment for the exclusion of HA and the exclusive reliance on post-ACTH cortisol in definitive testing protocols.

## Data Availability

If required, the raw data supporting the conclusions of this article will be made available by the authors, without undue reservation.

## References

[1] Van Lanen K Sande A. Canine hypoadrenocorticism: pathogenesis, diagnosis, and treatment. Top Companion Anim Med. (2014) 29:88–95. doi: 10.1053/j.tcam.2014.10.001, PMID: 25813848

[2] Guzman Ramos PJ Bennaim M Shiel RE Mooney CT. Diagnosis of canine spontaneous hypoadrenocorticism. Canine Med Genet. (2022) 9:6. doi: 10.1186/s40575-022-00119-4, PMID: 35505424 PMC9066729

[3] Klein SC Peterson ME. Canine hypoadrenocorticism: part I. Can Vet J. (2010) 51:63–9. 20357943 PMC2797351

[4] Peterson ME Kintzer PP Kass PH. Pretreatment clinical and laboratory findings in dogs with hypoadrenocorticism: 225 cases (1979-1993). J Am Vet Med Assoc. (1996) 208:85–91. doi: 10.2460/javma.1996.208.01.85, PMID: 8682712

[5] Bovens C Tennant K Reeve J Murphy KF. Basal serum cortisol concentration as a screening test for hypoadrenocorticism in dogs. J Vet Intern Med. (2014) 28:1541–5. doi: 10.1111/jvim.12415, PMID: 25066405 PMC4895569

[6] Gold AJ Langlois DK Refsal KR. Evaluation of basal serum or plasma cortisol concentrations for the diagnosis of Hypoadrenocorticism in dogs. J Vet Intern Med. (2016) 30:1798–805. doi: 10.1111/jvim.14589, PMID: 27714859 PMC5115184

[7] Lennon EM Boyle TE Hutchins RG Friedenthal A Correa MT Bissett SA. Use of basal serum or plasma cortisol concentrations to rule out a diagnosis of hypoadrenocorticism in dogs: 123 cases (2000-2005). J Am Vet Med Assoc. (2007) 231:413–6. doi: 10.2460/javma.231.3.413, PMID: 17669044

[8] Wakayama JA Furrow E Merkel LK Armstrong PJ. A retrospective study of dogs with atypical hypoadrenocorticism: a diagnostic cut-off or continuum? J Small Anim Pract. (2017) 58:365–71. doi: 10.1111/jsap.12649, PMID: 28247992 PMC5496775

[9] Hauck C Schmitz SS Burgener IA Wehner A Neiger R Kohn B. Prevalence and characterization of hypoadrenocorticism in dogs with signs of chronic gastrointestinal disease: a multicenter study. J Vet Intern Med. (2020) 34:1399–405. doi: 10.1111/jvim.15752, PMID: 32573832 PMC7379021

[10] Klein SC Peterson ME. Canine hypoadrenocorticism: part II. Can Vet J. (2010) 51:179–84. 20436864 PMC2808283

[11] Robinson NJ Brennan ML Cobb M Dean RS. Common decisions made and actions taken during small-animal consultations at eight first-opinion practices in the United Kingdom. Prev Vet Med. (2017) 139:1–9. doi: 10.1016/j.prevetmed.2016.12.002, PMID: 28364827

[12] Nemeth K Sterczer A Kiss DS Lanyi RK Hemzo V Vamos K. Determination of bile acids in canine biological samples: diagnostic significance. Meta. (2024) 14:1–35. doi: 10.3390/metabo14040178, PMID: 38668306 PMC11052161

[13] Pena-Ramos J Barker L Saiz R Walker DJ Tappin S Hare CHZ. Resting and postprandial serum bile acid concentrations in dogs with liver disease. J Vet Intern Med. (2021) 35:1333–41. doi: 10.1111/jvim.16134, PMID: 33955592 PMC8163115

[14] Boretti FS Meyer F Burkhardt WA Riond B Hofmann-Lehmann R Reusch CE. Evaluation of the cortisol-to-ACTH ratio in dogs with Hypoadrenocorticism, dogs with diseases mimicking Hypoadrenocorticism and in healthy dogs. J Vet Intern Med. (2015) 29:1335–41. doi: 10.1111/jvim.13593, PMID: 26250121 PMC4858040

[15] Lippi G Guidi GC Mattiuzzi C Plebani M. Preanalytical variability: the dark side of the moon in laboratory testing. Clin Chem Lab Med. (2006) 44:358–65. doi: 10.1515/CCLM.2006.073 16599826

[16] Wallin O Soderberg J Van Guelpen B Stenlund H Grankvist K Brulin C. Blood sample collection and patient identification demand improvement: a questionnaire study of preanalytical practices in hospital wards and laboratories. Scand J Caring Sci. (2010) 24:581–91. doi: 10.1111/j.1471-6712.2009.00753.x, PMID: 21050248

[17] Johnson CM Kass PH Cohen TA Feldman EC. Effect of intravenous or perivascular injection of synthetic adrenocorticotropic hormone on stimulation test results in dogs. J Vet Intern Med. (2017) 31:730–3. doi: 10.1111/jvim.14708, PMID: 28407319 PMC5435047

